# A Case of Atypical Mucin Balls Wearing Extended Wear of Silicone Hydrogel Lens for Therapeutic Use

**DOI:** 10.1155/2013/167854

**Published:** 2013-04-27

**Authors:** Yusuke Matsuzaki, Hiroshi Toshida, Toshihiko Ohta, Akira Murakami

**Affiliations:** ^1^Department of Ophthalmology, Juntendo University Shizuoka Hospital, 1129, Nagaoka, Izunokuni, Shizuoka 410-2295, Japan; ^2^Department of Ophthalmology, Juntendo University School of Medicine, 2-1-1, Hongo, Bunky0-ku, Tokyo 113-8421, Japan

## Abstract

A 25-year-old man visited our hospital showing atopic conjunctivitis and corneal shield ulcer on his left eye. Although eye drops of 0.1% of betamethasone sodium phosphate and 0.1% of hyaluronic acid ophthalmic solution were prescribed, calcific corneal opacities developed. The corrected visual acuity decreased to 6/20 in Snellen chart. After corneal epithelial exfoliation, removal of calcific corneal opacity was scrubbed with MQA soaked in 0.05 M of ethylenediaminetetraacetic acid (EDTA). After washing the eye with 200 mL of physiological saline, a silicon hydrogel lens, PureVision (balafilcon A), was inserted to obtain pain relief for the therapeutic use. At postoperative day 11, mucin balls were found between cornea and contact lens and stained by rose bengal dye. One of them was atypically larger than usual, and the major axis was approximately 1.5 mm. Wearing lens was stopped, and all of mucin balls and corneal staining were disappeared at postoperative day. Little corneal opacity remained, and visual acuity after surgery recovered to 14/20 at five months.

## 1. Introduction

Silicone hydrogel lenses show very high oxygen permeability. Although its safety and efficacy are well known, the development of mucin balls is reported in extended wearers of silicone hydrogel lenses [1–5]. We report a case with atypical mucin balls during extended wear of silicone hydrogel lens developed after keratectomy for calcific corneal opacities.

## 2. Case Report

**Figure 1:**
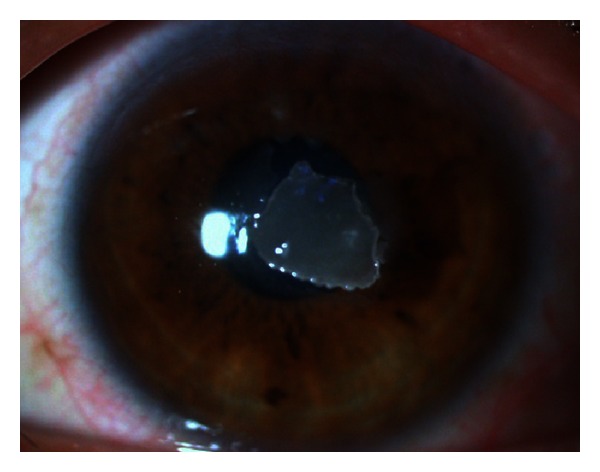
Slitlamp microscopic image of a 25-year-old man showing corneal shield ulcer at 40 days after beginning of treatment. After diminishing atopic conjunctivitis and corneal shield ulcer, calcific corneal opacities developed under the corneal epithelium.

A 25-year-old man visited our hospital with ocular pain on the left eye, showing corneal shield ulcer and atopic conjunctivitis. Eye drops of 0.1% of betamethasone sodium phosphate and 0.1% of hyaluronic acid ophthalmic solution were prescribed, but his ocular pain and corneal findings persisted. The corrected visual acuity was 6/20 in Snellen chart at the first visit. At 40 days after the beginning of treatment, calcific corneal opacities developed in the corneal epithelium. Six months after the first visit, the corrected visual acuity decreased to 4/20, and keratectomy was planned to remove calcification in the cornea (Figure 1).

The operative procedure included corneal epithelial exfoliation over almost the entire cornea using a golf club spud, followed by removal of corneal opacity using MQA (MQA eco-stick, Inami, Tokyo, Japan) soaked in 0.05 M of ethylenediaminetetraacetic acid (EDTA) (Sigma-Aldrich, St. Louis, MO, USA) [6]. After washing the eye with 200 mL of physiological saline, a silicon hydrogel lens, PureVisionTM (balafilcon A) (Bausch & Lomb, Rochester, NY, U.S.), was inserted to obtain pain relief for the therapeutic use. We have got obtain permission by ethics committee of our hospital for this procedure. Postoperative medication included instillation of levofloxacin ophthalmic solution, 0.1% fluorometholone ophthalmic solution, and 0.1% hyaluronic acid ophthalmic solution 4 times a day for 2 weeks. Ofloxacin eye ointment was applied twice daily for only 4 days. At the end of the surgery, a silicone hydrogel lens was inserted continuously for 1 week to reduce ocular pain.

**Figure 2:**
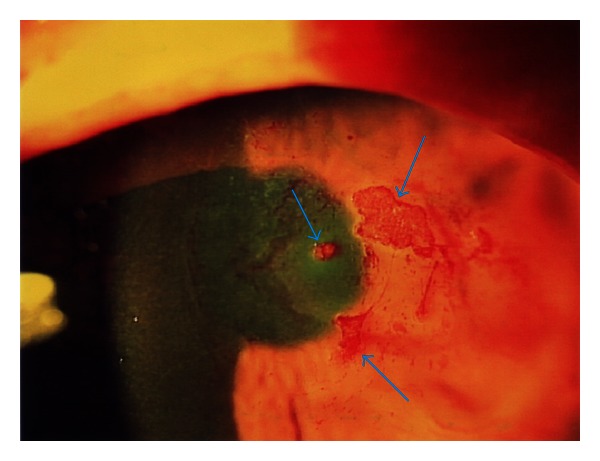
Atypically larger mucin balls found at post-operative day 11 wearing silicone hydrogel lens. They were stained with rose bengal dye (arrows).

**Figure 3:**
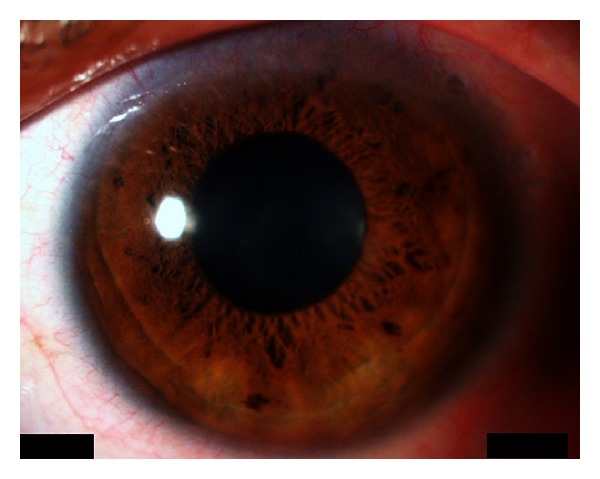
Corneal image at the final visit at 5 months after surgery. Little opacity was found in the cornea.

At postoperative day 11, wearing second lens after post-operative day 7, mucin balls were found between cornea and contact lens and were stained with rose bengal eye solution. One of them was atypically larger than usual size, which could vary between about 20 and 200 *μ*m. In the present case, the major axis was approximately 1.5 mm (Figure 2). At the same time, as corneal erosion was diminished, wearing silicone hydrogel lens was stopped and instillation of every eye drop was continued until the disappearing of all the corneal staining. One week later (at post-operative day 18), all of mucin balls and corneal staining were disappeared and the corrected visual acuity increased to 6/20. Little corneal opacity remained, and visual acuity after surgery recovered to 14/20 at five months (Figure 3). There was no change between preoperative corneal endothelial cell density (2797.5 ± 100.8 cells/mm^2^) and postoperative (2840.6 ± 123.1 cells/mm^2^).

## 3. Discussions

Contact lenses are not only used for correcting refractive errors but also for therapeutic purposes in the treatment of corneal epithelial disorders, ocular surface protection, and alleviation of pain [7, 8]. Theoretically, silicone hydrogel lenses show high oxygen permeability and can be worn continuously for one week or one month [9, 10]. The incidence of corneal ulceration due to silicone hydrogel contact lenses is comparable to that caused by conventional soft contact lenses, so silicone hydrogel lenses are thought to be safer [11]. These advantages have raising expectations about the therapeutic use of soft contact lens for a bandage effect, and the efficacy and safety of such use have been also reported. In the present case, although silicone hydrogel lens was used for the same purpose after keratectomy for calcific corneal opacities, mucin balls were developed. 

Mucin balls are a collection of particulate matter located between cornea and contact lens. It is thought to be related to the hardness of the lens material because mucin ball is generally found in patients with comparatively harder lenses, including silicone hydrogel lenses. This might be one of the reasons for the appearance of mucin balls in the present case. The size of mucin balls in the present case was larger than the previous report. So, it might be an atypical mucin balls. There is another possibility that severe conjunctival papillae compressed the cornea and the tear fluid over contact lens. That stress may result in developing mucin balls. The influence of ophthalmic solutions administered postoperatively on lens morphology is another factor to consider.

In addition, there is a possibility that corneal calcium deposition in this case might be the instillation of topical steroid phosphate preparations as in the previous report [12]. And when corneal erosion was disappeared, we defined that extended wear of contact lens was not necessary. After that, mucin balls were disappeared. To stop wearing extended silicone hydrogel lens might be effective to treat mucin balls including atypical types like the present case.
